# What Is the Real Clausius Statement of the Second Law of Thermodynamics?

**DOI:** 10.3390/e21100926

**Published:** 2019-09-24

**Authors:** Ti-Wei Xue, Zeng-Yuan Guo

**Affiliations:** Key Laboratory for Thermal Science and Power Engineering of Ministry of Education, Department of Engineering Mechanics, Tsinghua University, Beijing 100084, China; xtw18@mails.tsinghua.edu.cn

**Keywords:** second law of thermodynamics, Clausius Statement, theorem of the equivalence of transformations

## Abstract

In this paper, we first analyze the difference between the second law of thermodynamics and the laws in other disciplines. There are some phenomena in other disciplines similar to the Clausius Statement of the second law, but none of them has been accepted as the statement of a certain law. Clausius’ mechanical theory of heat, published in the nineteenth century, is then introduced and discussed in detail, from which it is found that Clausius himself regarded “Theorem of the equivalence of the transformation of heat to work, and the transformation of heat at a higher temperature to a lower temperature”, rather than “Heat can never pass from a colder to a warmer body without some other change”, as the statement of the second law of thermodynamics. The latter is only laid down as the fundamental principle for deriving the theorem of the equivalence of transformations. Finally, based on the theorem of the equivalence of transformations and the average temperature method, a general quantitative relation among the heat, the work, and the temperatures is obtained for arbitrary cycles, which is thus recommended as an alternative mathematic expression of the second law. Hence, the theorem of the equivalence of transformations is the real Clausius Statement of the second law of thermodynamics.

## 1. Introduction

As with the first law of thermodynamics, the second law of thermodynamics has been verified by countless natural facts. The second law has different statements for different physical phenomena. The classic statements given in most of the literatures mainly include the Clausius Statement and the Kelvin–Planck Statement [1,2,3,4,5]. The Clausius Statement was expressed as “Heat can never pass from a colder to a warmer body without some other change, connected therewith, occurring at the same time”, and the Kelvin–Planck Statement as “It is impossible to construct a device that operates in a cycle and produces no other effect than the production of work and the transfer of heat from a single body”. The Clausius Statement is more in accord with experience and thus easier to accept, while the Kelvin–Planck Statement provides a more effective means for bringing out second law deductions related to a thermodynamic cycle [3]. Besides, the Carathéodory Statement also occupies an important position in thermodynamics, which is “In the neighbourhood of any arbitrary state of a thermally isolated system, there are states which are inaccessible from that state” [6,7]. It is not more widely used but brings out its essential features in a way that the traditional treatment does not [7]. Since then, various understandings for the statements of the second law came out continuously [8,9,10,11], which help to clarify the connotation of the second law from different angles. It is important to emphasize that these statements are logically interlinked, consistent, and equivalent [5,12,13].

However, it is strange that the second law of thermodynamics is quite different from other laws, like Newton’s law of motion, Ohm’s law of electric conduction, and Fourier’s law of heat conduction, etc. There are differences such as; (1) there are many different statements for the same law; (2) it is only a qualitative description of a physical phenomenon, rather than a quantitative relationship between different physical quantities; and (3) some phenomena similar to the Clausius Statement exist in other disciplines. For example, an object can never move from low to high location in a gravity field without some other change, and electrical charges can never move from low to high potential in an electrostatic field without some other change. However, none of them has been accepted as the statement of a certain law. With that in mind, we have reviewed Clausius’ mechanical theory of heat, published in the nineteenth century [14,15], and indeed found that he himself only called “Heat can never pass from a colder to a warmer body without some other change, connected therewith, occurring at the same time” a fundamental principle rather than the statement of the second law of thermodynamics.

## 2. Misunderstanding of the Clausius Statement

### 2.1. Carnot’s Theorem

Before discussing the Clausius Statement, Carnot’s work must be first introduced because it is the cornerstone of the second law of thermodynamics [16,17,18,19].

Combined with engineering practice experience, Carnot realized that to keep a steam engine running, the working fluid does not only have to absorb heat from the boiler, but also has to release heat to the cooling water in the “condenser” [20]. In other words, when work is generated between two heat reservoirs, there must be a certain amount of heat flowing from the warmer reservoir to the cooler reservoir. The idea of circulation first formed in Carnot’s mind [20]. Based on the analogies of “fall of caloric” and “fall of water”, Carnot further realized that a certain reversible heat engine cycle always provides the same thermal efficiency for any working fluid. That is to say, the heat transport and the work production obey a certain relationship independent of the nature of the working fluid [15], which is exactly Carnot’s theorem.

However, this model does not completely agree with current perceptions [14,15,17,18,21]. Carnot did not yet know the first law of thermodynamics, which states that, when work is produced, the same amount of heat has to be consumed in a reversible cycle. As a result, only a part of the heat is released to the colder body, which is less than the heat from the warmer body. In other words, work cannot be created out of nothing. At that time, thermal efficiency was very low, so the amount of heat absorbed was almost the same as the amount of heat released [20]. In addition, at that time, the backward measurement tools had large measurement errors. Therefore, Carnot could not find the quantity difference between the two parts of heat.

On the positive side, Carnot wondered how the location of the temperature range affects thermal efficiency [22]. He discovered that a given fall of caloric (with a given temperature difference) produces more motive power at inferior than at superior temperatures. In his notation, the thermal efficiency was given by:

(1) e=F′(t)dt

where *F′*(*t*) is a universal function that was called the Carnot function afterwards and *t* represents the temperature. Unfortunately, because of the cognitive error about heat at that time, Carnot could not determine this function, so he did not know the maximum value of thermal efficiency, not even for an infinitesimal cycle [22]. The Carnot function, however, partly because of its universal character, provided a strong stimulus for further research on the subject. The problem was left open for Clausius to solve twenty five years after Carnot [22].

Even so, Carnot’s groundbreaking statement that “The relation between the falling heat and the produced work is independent of the nature of working fluid” is still correct. This laid a foundation for the development of the second law of thermodynamics.

### 2.2. Clausius Statement

Clausius found early on that Carnot’s work about the second law was incomplete and vague [14,15]. He wanted the law of conversion of heat to work in a thermodynamics cycle.

Considering the natural tendency that heat always passes from a warmer to a colder body for eliminating the temperature difference, Clausius presented a well-known proposition that “Heat can never pass from a colder to a warmer body without some other change, connected therewith, occurring at the same time”. Unfortunately, it met with much opposition [15]. Muller [22] stated that “this statement, suggestive though it is, has often been criticized as vague”. And Muller believed that Clausius himself did not also feel entirely satisfied with this proposition for the reason of it lacking an unequivocal mathematic description [22]. We further find that this proposition had only been laid down as a fundamental principle by Clausius, according to his original literature, “The Mechanical Theory of Heat” [14,15]. In spite of this, regrettably, ever since then, this fundamental principle was usually mistaken for the Clausius Statement of the second law, probably because of its well-understood empirical property.

After Joule’s heat-work equivalence theorem was found, Clausius corrected Carnot’s theorem successfully [21]. The relation of the heat and the work is still independent of the nature of the working fluid in a reversible cycle, but the heat from the hot source is not the same as the heat going into the cold source, and the difference between them is just the work. In order to get the law of conversion of heat to work, he further considered a reversible thermodynamic cycle as a combination of two kinds of transformations, that is, the transformation of heat to work and the transformation of heat at a higher temperature to a lower temperature [14,15,21]. He thought that “Carnot’s theorem actually expressed a relation between the two kinds of transformations, which may be regarded as phenomena of the same nature and are then equivalent*”* [14]. He called it the theorem of the equivalence of transformations [14,15].

**Figure 1:**
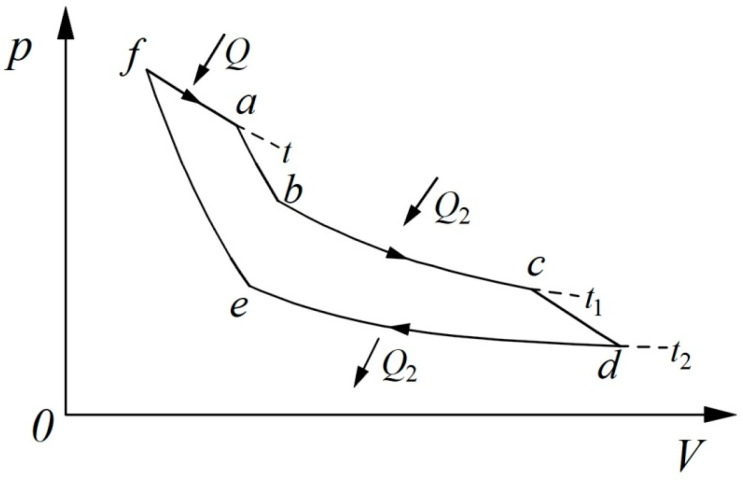
*p-V* diagram of a thermodynamic cycle with three heat reservoirs.

With that in mind, Clausius quantified the two kinds of transformations by giving them “Equivalence-Values”. He employed a thermodynamic cycle made up of three isothermal processes and three adiabatic processes [14,15], as shown in Figure 1. Processes f~a, b~c, and d~e are isothermal processes with temperatures *t*, *t*_1_, and *t*_2_ and heat quantities *Q*, *Q*_2_, and *Q*_2_, respectively. The other processes are adiabatic processes.

Since the heat quantity *Q*_2_ in process b~c is equal to the heat quantity *Q*_2_ in process d~e, the combination of the two processes could be equivalent to the transformation of heat from a high temperature to a lower temperature, and, thus, process f~a is equivalent to the transformation of heat to work. Clausius considered the transformation of work to heat and, therefore, the transformation of heat from a higher temperature to a lower temperature, as being positive. For the transformation of heat to work, the Equivalence-Value must be proportional to the heat quantity, *Q,* and must also depend on the temperature, *t*, which was therefore represented by −*Qf*(*t*) [14,15]. In a similar manner, the Equivalence-Value for the heat transformed from a higher temperature to a lower temperature must be proportional to *Q*_2_ and must also depend on the two temperatures *t*_1_ and *t*_2_, which was expressed by *Q*_2_*F*(*t*_1_,*t*_2_) [14,15]. The two transformations must be equal in magnitude. For the reversible thermodynamic cycle, the sum of the two Equivalence-Values must be zero:

(2) $$-Qf\left(t\right)+Q_{2}F\left(t_{1},t_{2}\right)=0$$

It is understood that the two transformations in a reversible cycle cancel each other out. Equation (2) is the mathematic expression of the theorem of the equivalence of transformations. Clausius had pointed out that the second law of thermodynamics was called the theorem of the equivalence of transformations in his mechanical theory of heat. Hence, the real Clausius Statement of the second law should be the theorem of the equivalence of transformations.

Harvey [23] stated that Clausius’ analysis was brilliant, but the idea of equivalence of transformations is difficult to grasp and is not even mentioned in most thermodynamics textbooks. However, the equivalence of transformations is, we think, of momentous significance for the second law of thermodynamics, as with the equivalence of work and heat for the first law of thermodynamics. The theorem of the equivalence of transformations is a basic theory of classical thermodynamics and it is necessary to be fully aware of it. In the next section, we will see that the Clausius Equality/Inequality and the concept of entropy came from the theorem of the equivalence of transformations [24].

## 3. Alternative Mathematic Expression of the Second Law

### 3.1. Clausius Equality/Inequality and Entropy

When *t* in the cycle in Figure 1 changes to *t’* and *Q* thus changes to *Q’*, the following relation appears [14]:

(3) $$-Q\prime f\left(t\prime \right)+Q_{2}F\left(t_{1},t_{2}\right)=0$$

If *t’* is greater than *t*, then let the thermodynamic cycle, including the temperature, *t*, work in reverse. The superposition of the two thermodynamic cycles will produce a new Carnot cycle with temperatures *t* and *t’*. Then Equation (2) and Equation (3) lead to:

(4) $$\frac{Q}{Q\prime }=\frac{f\left(t\prime \right)}{f\left(t\right)}$$

In this new Carnot cycle, *Q’* can be further divided into two parts; *Q’-Q* and *Q*. The first part is regarded as the heat transformed to work and the second part as the heat transformed from a higher temperature to a lower temperature. The following equation can be obtained as with Equation (3):

(5) −(Q′−Q)f(t′)+QF(t′,t)=0

Based on Equation (4) and Equation (5), the following relation is obtained:

(6) F(t′,t)=f(t)−f(t′)

Thus, the Equivalence-Value of the transformation of heat from a higher temperature to a lower temperature can be expressed by:

(7) QF(t′,t)=Qf(t)−Qf(t′)

According to Equation (7), the transformation of heat from a higher temperature to a lower temperature is equivalent to the combination of two opposite transformations between heat and work, that is, heat is transformed to work at *t*’, followed by work being transformed to heat at *t*. Bailyn [21] thus pointed out that, in this sense, the transformation between heat and work is the fundamental transformation. Therefore, for a process with a series of continuous heat reservoirs, the sum *N* of the Equivalence-Values of all the transformations will be:

(8) N=∫f(t)dQ

For a reversible cycle, it can be proved by the fundamental principle that all the transformations must cancel each other out (please refer to Clausius’ mechanical theory of heat for details). Thus, the sum of the Equivalence-Values of all the transformations in a reversible cycle must be zero. The integrating factor *f*(*t*) is just the reciprocal temperature,1/*T*, that is unique and guaranteed to be a valid integrating factor by the second law [25]. Now we come to the equality:

(9) $$\oint \frac{dQ}{T}=0$$

Clausius called Equation (9) the mathematic expression of the second law of thermodynamics [14,15], which was later named the Clausius Equality.

According to the state axioms, d*Q*/*T* must be the differential of a certain state quantity, which is just entropy, *S*, that Clausius first discovered, whose differential definition is thus:

(10) $$dS=\frac{dQ}{T}$$

For an irreversible thermodynamic cycle, the algebraic sum of all transformations can only be positive [14]. Hence there is an inequality:

(11) $$\oint \frac{dQ}{T}<0$$

where the heat released into a heat reservoir from the working fluid is defined as negative. Afterwards, Equation (11) was called the Clausius Inequality.

The Clausius Equality predicts the entropy flow balance in a reversible cycle, while the Clausius Inequality shows the non-conservation of entropy flow in an irreversible cycle. The Clausius Equality/Inequality, ∮δQ/T≤0, that is the combination of Equations (9) and (11) has been widely believed to be the mathematic expression of the second law of thermodynamics [21].

### 3.2. Alternative Mathematic Expression of the Second Law

As Section 2.1 mentions, Carnot did not find the maximum value of the thermal efficiency because the Carnot function was unknown. Fortunately, Clausius identified the Carnot function and further obtained the thermal efficiency of a Carnot cycle in terms of the theorem of the equivalence of transformations.

**Figure 2:**
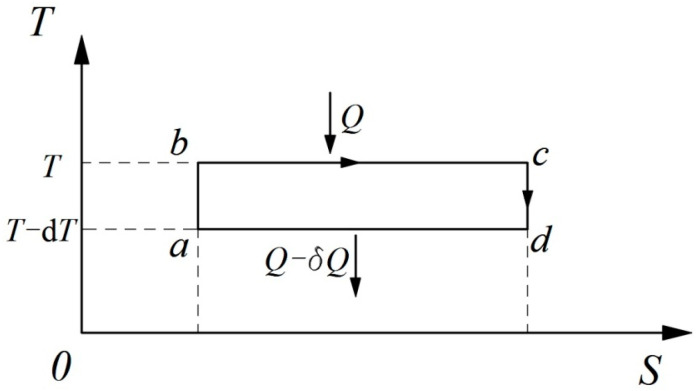
*T-S* diagram of an infinitesimal Carnot cycle.

For an infinitesimal Carnot cycle, as shown in Figure 2, from the theorem of the equivalence of transformations we can directly derive:

(12) $$-\frac{Q}{T}+\frac{Q-\delta Q}{T-dT}=0$$

Thus, the expression of the thermal efficiency of a Carnot cycle is:

(13) $$e=\frac{\delta Q}{Q}=\frac{1}{T}dT=\frac{1}{T}dt$$

Comparing Equation (13) with Equation (1), given by Carnot, the Carnot function can be obtained:

(14) $$F\prime (t)=\frac{1}{T}$$

So the Carnot function is just the reciprocal of the absolute temperature [14,22]. There is no doubt that Clausius is the first person to determine the Carnot function and the thermal efficiency of a Carnot cycle [22].

For a Carnot cycle with two different heat source temperatures, the thermal efficiency can also be derived directly based on the theorem of the equivalence of the transformations. The cycle, as shown in Figure 1, is reconsidered. Because the work quantity, *W*, is equal to the heat quantity, *Q*, the Equivalence-Value of transformation of heat to work can be rewritten as −*W/T*. The Equivalence-Value for the heat transformed from the high temperature, *T*_1_, to the low temperature, *T*_2_, is *Q*_2_ (1/*T*_2_ − 1/*T*_1_). The sum of Equivalence-Values of all transformations in the cycle is zero:

(15) $$-\frac{W}{T}+Q_{2}(\frac{1}{T_{2}}-\frac{1}{T_{1}})=0$$

When *T* changes to *T*_1_, the cycle becomes a Carnot cycle. Thus Equation (15) becomes:

(16) $$-\frac{W}{T_{1}}+Q_{2}(\frac{1}{T_{2}}-\frac{1}{T_{1}})=0$$

After rearranging Equation (16), we can obtain the expression of thermal efficiency:

(17) $$\eta =\frac{W}{Q_{1}}=1-\frac{T_{2}}{T_{1}}$$

which can also be rewritten as a quantity relation between the heat, the work, and the two heat reservoir temperatures:

(18) $$W=Q_{1}(1-\frac{T_{2}}{T_{1}})$$

**Figure 3:**
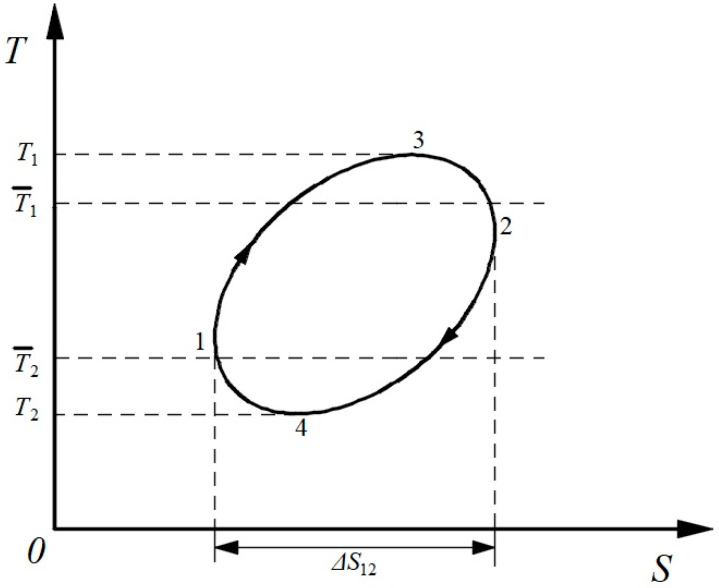
*T-S* diagram of an arbitrary reversible cycle.

However, for an arbitrary reversible cycle with multiple heat reservoirs, as shown in Figure 3, Equation (18) is no longer valid [26]. One solution to this problem is to employ “the average temperature method” to describe the heat absorption and release processes [3,27,28]. The average temperature method was first proposed by Alefeld [27]. The average temperature, $\bar{T}$, called the entropic average, was defined such that the entropy transfer would be the same if the entire heat transfer occurred at the average temperature [28]:

(19) $$\bar{T}=\frac{Q}{\Delta S}=\frac{Q}{\int \frac{1}{T}dQ}$$

This formulation retains the simplicity of the constant temperature model but makes it applicable to varying temperature processes [28]. Regarding entropy as a variable, the average temperature can be also expressed for a reversible process:

(20) $$\bar{T}=\frac{Q}{\Delta S}=\frac{\int TdS}{\Delta S}$$

Thus, the heat, *Q*, can be represented by the area under the curve in the *T-S* diagram. As shown in Figure 3, the entropy value of the working fluid is minimum at point 1 and maximum at point 2. Therefore, process 1-3-2 is the heat absorption process, while process 2-4-1 is the heat release process. The average temperature of the heat absorption process and the average temperature of the heat release process are respectively in an arbitrary reversible cycle:

(21) $$\left\{ \begin{array}{l} {\bar{T}}_{1}={\left(\frac{Q}{\Delta S}\right)}_{1-3-2}=\frac{\int_{1-3-2}TdS}{\Delta S_{12}}=\frac{Q_{1}}{\Delta S_{12}}\\ {\bar{T}}_{2}={\left(\frac{Q}{\Delta S}\right)}_{2-4-1}=\frac{\int_{1-4-2}TdS}{\Delta S_{12}}=\frac{Q_{2}}{\Delta S_{12}} \end{array}\right.$$

where ∆*S*_12_ is the total entropy change in the heat absorption process or release process, always defined as positive, *Q*_1_ represents the entire exchanged heat of the heat absorption process, equal to the area under the curve 1-3-2 to the *S*-axis, and *Q*_2_ represents the entire exchanged heat of the heat release process, equal to the area under the curve 2-4-1 to the *S*-axis. Thus, the quantity relation between the heat and the work for an arbitrary reversible cycle can be represented as:

(22) $$W=Q_{1}\left(1-\frac{Q_{2}}{Q_{1}}\right)=Q_{1}\left(1-\frac{{\bar{T}}_{2}\Delta S_{12}}{{\bar{T}}_{1}\Delta S_{12}}\right)=Q_{1}\left(1-\frac{{\bar{T}}_{2}}{{\bar{T}}_{1}}\right)$$

Equation (22), with the same form as Equation (18), is universally available to any reversible cycle.

As Section 3.1 mentioned, for an irreversible cycle, the algebraic sum of all transformations can only be positive. Therefore, the quantity relation between the heat and the work in an arbitrary irreversible cycle can be obtained:

(23) $$W<Q_{1}\left(1-\frac{{\bar{T}}_{2}}{{\bar{T}}_{1}}\right)$$

Finally, a general form of the quantity relation between the heat and the work for arbitrary cycles comes up:

(24) $$W\leq Q_{1}\left(1-\frac{{\bar{T}}_{2}}{{\bar{T}}_{1}}\right)$$

The mathematic formula of a science law usually expresses the quantitative relation among different physical quantities such as Ohm’s law and Fourier’s law. Equation (24) gives a universal quantitative relation between the heat and the work for any kind of cycle that the Clausius Equality/Inequality does not. Therefore, it could probably be perceived as an alternative mathematic expression of the second law of thermodynamics.

## 4. Conclusions

(1) Clausius had explicitly proclaimed in his mechanical theory of heat, published in the nineteenth century, that the second law of thermodynamics is “The theorem of the equivalence of transformations”, rather than “Heat can never pass from a colder to a warmer body without some other change”. The latter, as the description of a natural phenomenon, was only laid down as a fundamental principle. Similar natural phenomena also exist in other disciplines, none of which are yet accepted as the statements of a certain law. Hence, the theorem of the equivalence of transformations is the real Clausius Statement of the second law of thermodynamics.

(2) Precisely because of the theorem of the equivalence of transformations, the quantitative relationship between the heat and work in a Carnot cycle, as well as the Clausius Equality/Inequality, can be derived directly. Finally, the core physical quantity in thermodynamics, entropy, was discovered and defined.

(3) With the method of average temperature we summarize a general quantitative relation among the heat, the work, and the temperatures for any kind of cycles, $W\leq Q_{1}\left(1-\frac{{\bar{T}}_{2}}{{\bar{T}}_{1}}\right)$, from the theorem of the equivalence of transformations. It may be regarded as an alternative mathematic expression of the second law of thermodynamics.
